# Critical Review of Oncologic Medical Malpractice Claims Against Orthopaedic Surgeons

**DOI:** 10.5435/JAAOSGlobal-D-22-00169

**Published:** 2023-05-02

**Authors:** William Davis, Shravya Kichena, Michael D. Eckhoff, Benjamin R. Childs, Rajiv Rajani, Matthew E. Wells, Sean P. Kelly

**Affiliations:** From the Texas Tech University Health Sciences Center El Paso, El Paso, TX (Dr. Davis, Ms. Kichena, Dr. Eckhoff, Dr. Childs, and Dr. Wells); the Department of Orthopedic Surgery, William Beaumont Army Medical Center, El Paso, TX (Dr. Eckhoff, Dr. Childs, and Dr. Wells); the Department of Orthopedic Surgery, Texas Tech University Health Sciences Center El Paso, El Paso, TX (Dr. Eckhoff, Dr. Childs, Dr. Rajani, and Dr.Wells); and the Department of Orthopedic Surgery, Tripler Army Medical Center, Honolulu, HI (Dr. Kelly).

## Abstract

The purpose of this study was to determine the most common allegations for malpractice litigation brought against orthopaedic surgeons for oncologic matters and the resulting verdicts.

**Methods:**

The Westlaw Legal research database was queried for malpractice cases filed against orthopaedic surgeons for oncologic matters in the United States after 1980. Plaintiff demographics, state of filing, allegations, and outcomes of lawsuits were recorded and reported accordingly.

**Results:**

A total of 36 cases met the inclusion and exclusion criteria and were subsequently included in the final analysis. The overall rate of cases filed remained consistent through the past four decades and was primarily related to a primary sarcoma diagnosis in adult women. The primary reason for litigation was failure to diagnose a primary malignant sarcoma (42%) followed by failure to diagnose unrelated carcinoma (19%). The most common states of filing were primarily located in the Northeast (47%), where a plaintiff verdict was also more commonly encountered as compared with other regions. Damages awarded averaged $1,672,500 with a range of $134, 231 to $6,250,000 and a median of $918,750.

**Conclusion:**

Failure to diagnose primary malignant sarcoma and unrelated carcinoma was the most common reason for oncologic litigation brought against orthopaedic surgeons. Although most of the cases ruled in favor of the defendant surgeon, it is important for orthopaedic surgeons to be aware of the potential errors that not only prevent litigation but also improve patient care.

Medical liability is intrinsic to the practice of orthopaedic surgery, and awareness of prior claims may help surgeons avoid similar pitfalls. Medical malpractice generally involves a claim that a healthcare provider displayed professional negligence with substandard care and, ultimately, caused harm to a patient. Successful claims require four essential elements: (1) a duty established to provide care to the patient, (2) a breach in the standard of care, (3) the breach directly lead to injury of the patient, and (4) existence of lasting damages to the patient such that the legal system could provide redress.^1^ Although outcomes tend to favor defendants, a successful medical malpractice lawsuit awards the plaintiff a median amount of $1.2 million dollars (US) and continues to rise.^2^ Oncology-related malpractice claims, while uncommon, can be encountered by orthopaedic surgeons of any subspeciality.

The American Cancer Society projects there will be nearly two million new cancer diagnoses within the United States in 2022.^3^ Due to developments in systemic therapies, patients with carcinomas are living longer and are more likely to experience metastatic disease to bone and subsequent evaluation by an orthopaedic surgeon.^4^ Given this potential increase in complex patient encounters, orthopaedic surgeons may also have increased exposure to liability as represented by medical malpractice claims.

Examples of previous findings in orthopaedics surgery claims research include the following^5-7^: Arthroscopists are sued for vascular complications or wrong-sided surgery,^5^ spine surgeons for failing to obtain informed consent,^6^ and arthroplasty surgeons for infection and nerve injuries.^7^ However, there has been limited evidence focusing on oncologic malpractice claims specific to orthopaedic surgeons.^8^ Primary bone and soft tissue sarcomas collectively comprise an estimate less than 1% of all new cancer diagnoses in the United States,^3^ whereas carcinomas and the related care (including metastatic bone disease) represent a large potential source of orthopaedic surgeon encounters. The primary outcome of this study was to determine the most common allegations in litigation brought against orthopaedic surgeons for all cancers. Secondary outcomes were to dispose characteristics specific to each case, including plaintiff demographics, geographic region and year of filing, verdicts, and compensation for successful lawsuits.

## Methods

**Figure 1:**
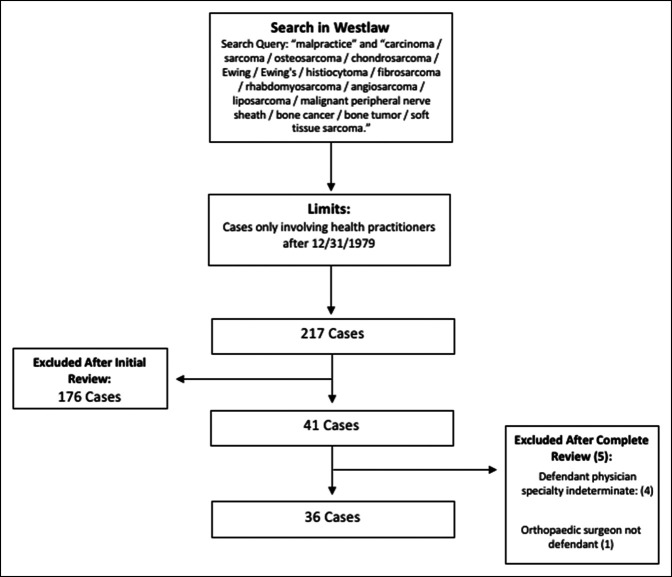
Flow diagram of case selection and stages of inclusion and exclusion.

Westlaw (Thomson Reuters) is an online legal database containing publicly available state and federal court records. As one of the largest collections of medical lawsuits, Westlaw has been used in previous research of medical malpractice claims within the United States.^5,6,7,8,9^ Each case recorded in the database contains pertinent plaintiff and defendant information as well as in-depth case summaries and outcomes. Westlaw was queried for litigation filed against orthopaedic surgeons for oncologic-related claims from 1980 to 2021. Search terms for sarcoma were based on the work of Hwang et al^8^ and included “malpractice” and “sarcoma/osteosarcoma/chondrosarcoma/Ewing/Ewing's/histiocytoma/fibrosarcoma/rhabdomyosarcoma/angiosarcoma/liposarcoma/malignant peripheral nerve sheath/bone cancer/bone tumor/soft tissue sarcoma.” Additional search terms included “malpractice” and “carcinoma/metastases/metastatic disease.” Plaintiff demographics to include age, sex, and cancer type were recorded. Case-specific factors including geographical region of filing, year of filing, allegation, verdict, and plaintiff awarded amounts were also extracted for additional analysis. The states of filing were categorized into five distinct geographic regions as defined by the National Geographic Society.^10^ Failure to diagnose as an allegation was subclassified based on the underlying diagnosis of primary malignant sarcoma, unrelated carcinoma, and benign lesions. Cases were excluded if physician specialty was indeterminate or if an orthopaedic surgeon was involved in the lawsuit but not as a defendant (Figure 1).

## Results

**Figure 2:**
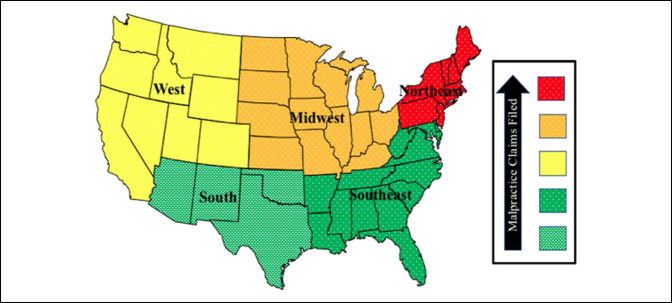
Diagram showing geographic distinction in oncology-related malpractice claims filed against orthopaedic surgeons.

A total of 36 cases met the inclusion and exclusion criteria and were included in the final analysis (Figure 1). Of these 36 cases, only 11% were filed against orthopaedic oncology specialists, while the remainder were filed against nononcologic specialists. The number of cases filed remained consistent through the past four decades, most of the cases were female patients, and the most common diagnosis was sarcoma (Table 1). The most common states of filing were in the Northeast (47%) followed by the Midwest (19%), West (17%), Southeast (11%), and Southwest (6%, Figure 2). The primary reason for litigation was the failure to diagnose a primary malignant sarcoma (42% of cases; Table 2). Failure to diagnose unrelated carcinoma, such as lung cancer, was the second-highest reason for litigation (19% of cases). A defense verdict occurred in 75% of cases, a plaintiff verdict in 17%, and a settlement in 3% (Table 3). The Northeast and Midwest had plaintiff verdict rates of 35% and 14%, respectively, while the Southeast, Southwest, and West had 0% plaintiff verdict rates. Damages awarded averaged $1,672,500 with a range of $134, 231 to $6,250,000 and a median of $918,750. No plaintiff verdicts or settlements were made in cases involving orthopaedic oncology specialists. A summary of case characteristics resulting in settlement or plaintiff verdict is provided in Table 4.

**Table 1:** Plaintiff-specific and Case-specific Characteristics Among Malpractice Claims Brought Against Orthopaedic Surgeons for Oncology-related Allegations From 1980-Present

Factor	No. of Cases Filed
Overall age of plaintiff	
Adult	31 (86%)
Minor	5 (14%)
Female plaintiffs	21 (58%)
Country region	
Northeast (*ME, VT, NH, MA, RI, CT, NJ, NY, PA*)	17 (47%)
Southeast (*MD, DE, DC, WV, VA, KY, TN, NC, SC, AR, LA, MS, AL, GA, FL*)	4 (11%)
Midwest (*ND, SD, NE, KS, MN, IA, MO, IA, WI, IL, IN, MI, OH*)	7 (19%)
Southwest (*AZ, NM, OK, TX*)	2 (6%)
West *(WA, OR, ID, MT, WY, CA, NV, UT, CO)*	6 (17%)
Cancer types	
Benign	4 (11%)
Sarcoma	17 (47%)
Carcinoma	7 (20%)
Not specified	8 (22%)
Year of case	
1980-1989	9 (25%)
1990-1999	8 (22%)
2000-2009	10 (28%)
2010-present	9 (25%)

**Table 2:** Allegations Leading to Oncologic-based Malpractice Claims Against Orthopaedic Surgeons From 1980-Present

Allegation	Overall No. of Cases	Percentage of Cases (%)
Failure to diagnose (*primary malignant sarcoma*)	15	42
Failure to diagnose *(unrelated carcinoma)*	7	19
Treatment error *(intraoperative complication)*	5	13
Treatment error *(intraoperative and postoperative complications)*	2	6
Incomplete informed consent	3	8
Failure to diagnose *(primary benign lesion)*	2	6
Failure to diagnose (*diagnostic biopsy error*)	1	3
Wrongful death	1	3

**Table 3:** The Verdicts Reported for Malpractice Claims Brought Against Orthopaedic Surgeons Between 1980-Present

Reason for Litigation	Defendant Verdict	Plaintiff Verdict	Settlement	Pending/Unreported	Total Available
Failure to diagnose (*primary malignant sarcoma*)	9	5	1	0	15
Failure to diagnose *(unrelated carcinoma)*	6	1	0	0	7
Treatment error (*intraoperative complication*)	5	0	0	0	5
Treatment error (*intraoperative and postoperative complications*)	2	0	0	0	2
Incomplete informed consent	2	0	0	1	3
Failure to diagnose (*primary benign lesion*)	2	0	0	0	2
Failure to diagnose (*diagnostic biopsy error*)	0	0	0	1	1
Wrongful death	1	0	0	0	1

**Table 4:** Summary of Case Characteristics Resulting in Settlement or Plaintiff Verdict

Year	Plaintiff Sex (M/F)	Tumor Type	Reason for Litigation	Awarded Amount	Description
1980	M	Fibrosarcoma	Failure to diagnose	$710,000	The plaintiff was initially treated by an orthopaedic surgeon for a traumatic upper extremity injury. On subsequent follow-up, patient's mother requested additional evaluation of an enlarging mass in the aforementioned arm. However, the surgeon reportedly documented this as a simple callus formation and provided no additional workup. The patient was eventually diagnosed with fibrosarcoma and required amputation of the involved arm.
1983	F	Ewing sarcoma	Failure to diagnose	Unknown	The plaintiff claimed the surgeon displayed gross negligence in their interpretation of radiographs resulting in failure to diagnose the underlying tumor in a timely manner.
1983	F	Not specified	Failure to diagnose	$1,237,500	The plaintiff continued to endorse pain after a foot surgery. The orthopaedic surgeon defendant stated it was likely due to a pinched nerve that would require surgical exploration and release; however, the patient refused additional treatment. The patient was eventually found to have malignant soft tissue sarcoma in the painful area.
1989	M	Adamantinoma	Failure to diagnose	$750,000	The plaintiff was treated for orthopaedic trauma; however, the surgeon failed to note or further investigate possible cancerous lesion on radiographs despite concerning radiologist reports. The patient was eventually diagnosed with adamantinoma that required surgical resection and additional care.
1993	F	Epithelioid sarcoma	Failure to diagnose	$6,250,000	The orthopaedic surgeon initially diagnosed a small mass on patient's ankle as a simple fibroma. The patient was later diagnosed with metastatic epithelioid sarcoma. There was also evidence regarding surgeon's alteration of patient's records, which resulted in $3,000,000 in punitive damages.
1994	F	Lung cancer	Failure to diagnose	$134, 231	The patient was treated for orthopaedic trauma; however, the surgeon failed to inform patient of possible lung carcinoma found on radiology reports before discharge. Patient was eventually diagnosed with lung carcinoma.
1995	M	Soft tissue sarcoma	Failure to diagnose	$1,087,500	The patient presented with a hamstring mass originally diagnosed as a muscular strain after trauma. On future reevaluations, the surgeon diagnosed the persistent mass as scar tissue. The patient was eventually diagnosed with a soft tissue sarcoma.

## Discussion

Overall, our study found that the most common reported oncologic allegations against orthopaedic surgeons were failure to diagnose sarcoma followed by failure to diagnose an incidental carcinoma. These results are consistent with the findings of Hwang et al^8^ in that delay in diagnosis was the primary reason for litigation for primary sarcoma when looking at providers across all specialities. In their analysis, orthopaedic surgeons comprised 23% of all cases. Our results similarly indicated that orthopaedic surgeons have claims filed for the evaluation of, rather than the surgery of, these lesions.

This seems to be consistent with trends seen in other malpractice suits. Failure to diagnose tends to be the primary reason for litigation among providers across various subspecialties in medicine.^11,12,13,14,15^ The primary plaintiff allegation was grounded in having delayed care and consequential progression of disease because of the initial failure to diagnose. Patients seem more likely to bring litigation against physicians who they felt failed to screen or arrange an appropriate referral for their cancer rather than the oncology specialists who then took over their treatment.

Most of the reported cases involved adult plaintiffs. Five cases included pediatric patients, with one case resulting in a plaintiff verdict. These results seem to be consistent with findings of previous literature showing that tumor-related matters comprise a very small percentage of pediatric orthopaedic malpractice claims and are more likely to be filed against nonorthopaedic specialties.^16^

Within this study, nearly 50% of total cases were filed in the Northeast. In addition, the Northeast had the highest plaintiff success rate of 35% when compared with the 0 to 14% success rates in other regions. High rates of medical malpractice occur in the Northeast in other orthopaedic subspecialties as well and are not an unexpected finding.^17^ In a similar analysis of orthopaedic lawsuits using Westlaw, the Northeast had the highest rates of litigation per capita with 8.7 cases filed per one million persons.^18^ One possible reason for this could be the perceived likelihood of winning a lawsuit in this locale. When looking at negligence laws, there are four categories (contributory, pure comparative, modified comparative, and slight-gross negligence comparative), with pure comparative being the most lenient toward plaintiffs. Under this approach, a plaintiff can be compensated for damages even if the plaintiff is more at fault than the defendant, increasing physician liability.^19^ On the other hand, pure contributory negligence is the most lenient toward physicians and does not award damages to the plaintiff if they contribute to their injuries in any way, even if the plaintiff only shares 1% of the fault. Most states that follow this law are located in the Southeast region which could explain why only 3 cases in this study came from this region and with a 0% rate of plaintiff verdicts. Medical malpractice noneconomic damage caps could also be contributing to these trends. Of the nine states included in the Northeast region, seven states have no cap limiting the amount of damages a plaintiff can expect to recover.^19^ This is a higher proportion of no-cap states than other regions (compared with one of four in the Southwest and four of nine states in the West). Therefore, the higher claim rates of the Northeast may be secondary to awareness of plaintiff-favoring legal structures, rather than the total number of oncology patients or differences in orthopaedic care.

Although most of the cases ruled in favor of the defendant surgeon, it is important for orthopaedic providers to be aware of the potential errors to not only prevent litigation but also improve patient care. Roughly 20% (7 cases) of the oncology-related lawsuits brought against orthopaedic surgeons were for failure to diagnose an unrelated carcinoma, most of which were lung cancer incidentally seen on imaging such as shoulder radiographs. However, only one of these cases was ruled in favor of the plaintiff. In that particular case, the orthopaedic surgeon neglected to read the radiology report mentioning a suspicious lung mass and was found at fault for not notifying the patient or conducting a workup. Of the six remaining cases that resulted in defendant verdicts, all involved similar failure to diagnose incidental cancer on imaging or failing to investigate a patient report with additional imaging. In addition, alteration of patient records by the orthopaedic surgeon would be ill advised (and illegal) because it resulted in an additional $3 million US in punitive damage on top of wrongful death and survivorship claims in one case. This was not the norm in this database query, and the most orthopaedic surgeons, even in litigation, did not alter or destroy records.

Although failure to diagnose seems to be the primary reason for litigation against orthopaedic surgeons for oncology-related matters, it also seems to be a very small percentage of total malpractice claims filed when compared with nononcology matters. Matsen et al^20^ reviewed more than 400 claims brought against orthopaedic surgeons through a medical liability insurer database and found only eight cases brought forth for failure to diagnose a neoplasm.

In plaintiff awarded amount, it would seem that oncology-related lawsuits against orthopaedic surgeons are similar to other reported estimates for non–oncology-related matters. Samuel et al^7^ reported an average plaintiff award amount of $1,433,874 with a range of $27,300 to $7,000,000 for litigation filed against orthopaedic surgeons after total joint arthroplasty. Of the cases ruling in favor of the plaintiff that disclosed the award or settlement amount, our study found an mean plaintiff award amount of $1,672,500 with a range of $134, 231 to $6,250,000. These findings suggest that although medical malpractice claims against orthopaedic surgeons for oncology-related matters are less likely, they can be just as costly.

There were additional reported allegations to include treatment errors, incomplete informed consent, and failure to diagnose due to diagnostic biopsy error. The seven cases of alleged treatment errors comprised two cases of postoperative fractures at tumor excision or biopsy sites, two cases of postoperative nerve damage, one case of adverse reaction to bone morphogenetic, one case of intraoperative organ damage, and one case of poor preoperative planning resulting in surgical delay and complications. In the three cases of alleged failure to provide complete informed consent, plaintiffs stated their orthopaedic surgeon did not provide all available treatment options or discuss postoperative risks. All of these reported treatment errors and improper consent cases resulted in defendant verdicts. Only one case was found regarding an error in performing a diagnostic biopsy. Mankin et al described biopsy-related diagnostic errors for bone and soft tissue tumors to be as high as 20% at the turn of the century.^21,22^ Many of these cases resulted with adverse outcomes to include need for amputation over standard limb salvage surgery. These types of cases may not have been captured by our database, were done by nonorthopaedic surgeons, or may not have resulted in litigation. Grossly negligent cases concerning biopsy error may also be settled outside of court entirely.

Interestingly, two litigation cases were filed against orthopaedic surgeons for failure to diagnose primary benign lesions. In both of these cases, patients initially presented with nonspecific knee pain diagnosed as patellar compression syndrome, and chondromalacia, however, was later diagnosed with giant cell tumors that required excision, reconstructive surgery, and postoperative complications. In both of these cases, the orthopaedic surgeons were found to have upheld reasonable standard of care in their initial assessments and diagnoses resulting in defendant verdicts.

Our study is not without limitations. Westlaw only reports cases submitted to its database and does not include cases settled out of court which may include grossly negligent, indefensible claims. In addition, in our effort to exclude nonorthopaedic surgeons, this could have possibly led to an underestimation of litigation claims and award amounts if the provider was not specified as an orthopaedic surgeon in the case summary. Finally, the specific specialty of the orthopaedic surgeon, unless stated in case documents or easily attained through publicly available physician profiles, was listed as a generalist. This could have potentially skewed the number of litigations brought against orthopaedic generalists as compared with fellowship-trained orthopaedic oncologists.

## Conclusion

Orthopaedic surgeons strive to provide exceptional care for all patients experiencing musculoskeletal disorders. Benign and malignant tumors are uncommon in general orthopaedic practice; however, they should always remain part of a differential diagnosis. Failure to diagnose benign, destructive tumors and unrelated carcinomas serves as the primary allegation among malpractice claims brought against orthopaedic surgeons. Obtaining timely, appropriate imaging and following up on the radiology reports are two simple ways to avoid malpractice claims in orthopaedic surgery. The Northeast region of the United States served as the most arduous legal landscape in both quantity of filed cases and the likelihood of plaintiff verdict. However, it is important to consider that certain regions, such as the Northeast, may perform more oncologic surgeries, thus increasing exposure and risk to litigation.^3^ Although most of the cases ruled in favor of the defendant surgeon, it is important for orthopaedic providers to be aware of potential errors that not only prevent litigation but also optimize patient care.
